# Considerations in the Identification of Endogenous Substrates for Protein L-Isoaspartyl Methyltransferase: The Case of Synuclein

**DOI:** 10.1371/journal.pone.0043288

**Published:** 2012-08-14

**Authors:** Gareth J. Morrison, Ranjani Ganesan, Zhenxia Qin, Dana W. Aswad

**Affiliations:** Department of Molecular Biology & Biochemistry, University of California Irvine, Irvine, California, United States of America; University of South Florida College of Medicine, United States of America

## Abstract

Protein L-isoaspartyl methyltransferase (PIMT) repairs abnormal isoaspartyl peptide bonds in age-damaged proteins. It has been reported that synuclein, a protein implicated in neurodegenerative diseases, is a major target of PIMT in mouse brain. To extend this finding and explore its possible relevance to neurodegenerative diseases, we attempted to determine the stoichiometry of isoaspartate accumulation in synuclein *in vivo* and *in vitro.* Brain proteins from PIMT knockout mice were separated by 2D electrophoresis followed by on-blot [^3^H]-methylation to label isoaspartyl proteins, and by immunoblotting to confirm the coincident presence of synuclein. On-blot ^3^H-methylation revealed numerous isoaspartyl proteins, but no signal in the position of synuclein. This finding was corroborated by immunoprecipitation of synuclein followed by on-blot ^3^H-methylation. To assess the propensity of synuclein to form isoaspartyl sites *in vitro*, samples of recombinant mouse and human α-synucleins were aged for two weeks by incubation at pH 7.5 and 37°C. The stoichiometries of isoaspartate accumulation were extremely low at 0.02 and 0.07 mol of isoaspartate per mol of protein respectively. Using a simple mathematical model based on the first order kinetics of isoaspartyl protein methyl ester hydrolysis, we ascribe the discrepancy between our results and the previous report to methodological limitations of the latter stemming from an inherent, and somewhat counterintuitive, relationship between the propensity of proteins to form isoaspartyl sites and the instability of the ^3^H-methyl esters used to tag them. The results presented here indicate that synuclein is not a major target of PIMT *in vivo*, and emphasize the need to minimize methyl ester hydrolysis when using methylation to assess the abundance of isoaspartyl sites in proteins.

## Introduction

**Figure 1:**
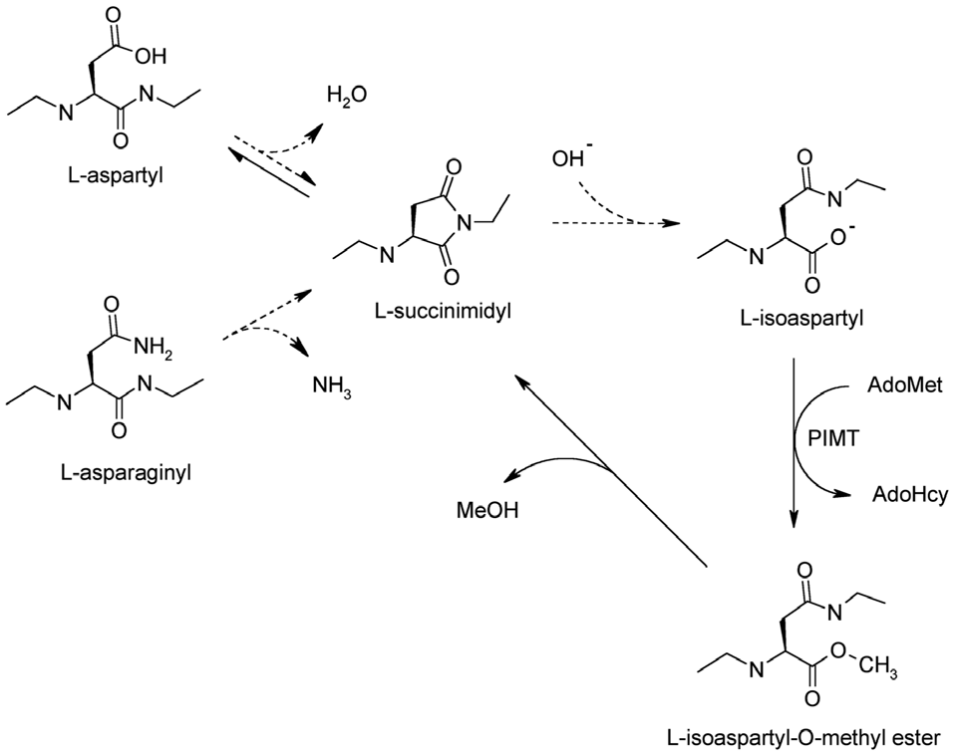
Mechanism of isoaspartate formation and PIMT catalyzed repair. Under physiological conditions, deamidation of asparaginyl residues or isomerization of aspartyl residues result in the formation of an intermediate succinimide. Upon spontaneous hydrolysis, a mixture of L-Asp (∼15–30%) and atypical L-isoAsp (∼70–85%) linkages are produced. Using AdoMet as a methyl donor, PIMT then selectively methylates the isoaspartyl α-carboxyl group to form a highly labile methyl ester. Spontaneous demethylation occurs within minutes to reform the original succinimide, with release of methanol as a by-product. This succinimide is now the starting point for further cycles of repair, resulting in near complete conversion of the isoaspartyl β-linkages to normal aspartyl α-linkages. Broken lines represent the degradative pathway, and solid lines represent the repair pathway.

Isoaspartate (isoAsp) formation, through deamidation of asparaginyl residues or isomerization of aspartyl residues, constitutes a large proportion of spontaneous protein damage observed both *in vivo* and *in vitro* [1]–[6]. Generation of isoAsp sites is initiated by nucleophilic attack on the Asx side-chain carbonyl by the C-flanking amide bond nitrogen resulting in an intermediate succinimide (Fig. 1). Hydrolysis of the succinimide generates mainly a mixture of α-linked L-aspartyl (∼15–30%) and β-linked L-isoaspartyl (∼70–85%) residues. Synthetic peptide studies have shown that altering the N+1 residue has a major influence on the propensity for isoAsp formation, with glycine, serine, and histidine most associated with “hot spots" of isoAsp formation. In structured proteins the same sequence effect is often found, but isoAsp formation is generally restricted to highly flexible regions of the polypeptide.

Protein L-isoaspartate *O*-methyltransferase (PIMT; EC 2.1.1.77), originally described as a methanol-forming enzyme in pituitary extracts [7], selectively methylates the α-carboxyl group of L-isoaspartyl residues [8], [9]. The isoAsp methyl ester formed is highly labile (T_1/2_≈7 min at pH 7.4, 37°C for -isoAsp-Gly-) and spontaneously demethylates to reform a succinimide that can restore the normal α-linked Asp-Xaa bond (Fig. 1) [10], [11]. Continuing cycles of PIMT action efficiently repair L-isoAsp sites, as has been demonstrated with a number of peptides and proteins [12]–[16]. A repair function for PIMT *in vivo* is supported by the observation that reduction of PIMT activity in cultured cells or (KO) knockout mice dramatically increases the level of isoAsp-containing proteins [17]–[20]. A critical need for PIMT action in the brain is evident by its high specific activity in this tissue [18], [21] as well as the overt neurological phenotype of PIMT KO mice: increased brain size, abnormal neuro-anatomical and electrophysiological properties of hippocampal cells, atypical behavior, and fatal epileptic seizures beginning at 4 weeks of age [18], [19], [22]–[24].

Identifying the major targets of PIMT-dependent repair should help explain how isoAsp formation alters brain function and its possible contribution to neurological disease and cognitive aging. In nuclear fractions of the PIMT-KO mouse brain, we found that histone H2B is a major substrate for PIMT [25], [26], suggesting that isoAsp formation may have a deleterious effect on gene expression. In a more recent study, we used a 2D gel-based proteomics approach to identify 22 PIMT substrates in post-nuclear extracts of the KO mouse brain [27]. Prominent among these were synapsins I and II, α- and β-tubulin, collapsin response mediator protein 2 (CRMP2), and dynamin-1. A parallel proteomic analysis of PIMT substrates in the KO mouse brain was carried out independently by the Carter group in the UK [28]. Major targets for PIMT reported in this latter study differed markedly from our study and included α- and β-synuclein. The absence of the synucleins (with masses of 14.5 and 14.0 kDa respectively) in our proteomic study was not surprising, as our second-dimension SDS-PAGE separation did not resolve proteins below 22 kDa.

**Figure 2:**
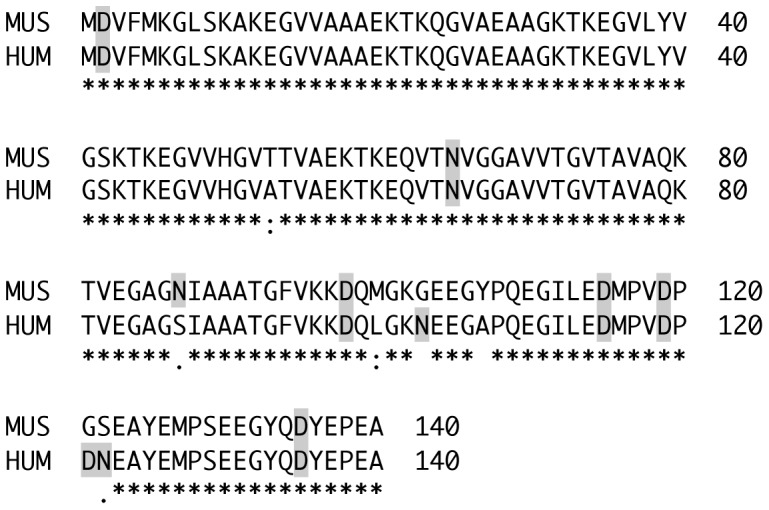
Protein sequence alignment of human (HUM; Swiss-Prot accession # P37840) and murine (MUS; Swiss-Prot accession #O55042) α-synuclein. All potential sites of isoAsp formation are highlighted. Note that 4 of the 7 sites of non-identity involve Asx residues.

The report of isoAsp formation in synuclein was of great interest to us given its presumed role in pre-synaptic function and because abnormal forms of synuclein are characteristic of Parkinson's and other neurological diseases. We noted also that mouse synucleins do not contain any of the predicted hotspot sequences typically associated with isoAsp formation (Fig. 2), suggesting that synuclein may contain one or more Asx residues conformationally poised for isoAsp formation at an atypical (non hot-spot) sequence, and thereby implying that isoaspartate in synuclein might have a functional role.

In the present study, we investigated the propensity of α- and β-synuclein to form isoAsp sites in the PIMT KO mouse model, and used *in vitro* aging of purified recombinant α-synuclein to compare the relative rates of isoAsp formation in mouse *vs* human sequences. Unlike the Vigneswara report [28], we were unable to detect any significant level of isoAsp accumulation in synuclein in the KO mouse brain. Moreover we found that when mouse and human α-synucleins are aged *in vitro*, they accumulate isoaspartyl sites at a rate that is about 10-fold slower than proteins (such as synapsin I and calmodulin) that are deemed to be highly susceptible to isoaspartate formation. To explain the discrepancies between the proteins identified in the Vigneswara proteomics study, *vs* our parallel proteomics study [27] and our present results with synuclein, we carried out a simple mathematical simulation that strongly suggests ^3^H-methyl tagging of isoaspartyl proteins prior to a lengthy 2D analysis (as employed by in the Vigneswara study) results in a preferential loss of ^3^H-tags on those proteins that are most susceptible to isoaspartate formation. In contrast, on-blot ^3^H-methylation, as employed in our recent studies, defers the tagging step until the 2D separation is completed and proteins have been blotted onto PVDF (polyvinylidene fluoride), resulting in an unbiased profile of those proteins that contribute the most to isoaspartate accumulation *in vivo.*

## Methods

### Ethics statement

All animal housing and procedures were performed using a protocol approved by the Institutional Animal Care and Use Committee of the University of California, Irvine (IACUC #: 2009–2882). Mice were anesthetized with a lethal dose of Euthasol® prior to decapitation.

### Materials

S-Adenosyl-[*methyl*-^3^H]-L-methionine ([^3^H] AdoMet) was purchased from PerkinElmer Life Sciences. IPG (immobilized pH gradient) strips (pH 4–7 and pH 3–10 NL; 7 cm lengths) and 2-D rehydration buffer were from Bio-Rad. Mouse anti-α/β synuclein (Syn 202), a monoclonal antibody raised against full-length human recombinant α/β synuclein, and protein A-agarose, were from Santa Cruz Biotechnology, Inc. Rabbit anti-pan synuclein polyclonal antibody and Immobilon-P PVDF membrane (0.45 µm) were purchased from Millipore. Rabbit anti-mouse CRMP2 polyclonal antibody was custom made by AnaSpec. Secondary antibody (HRP-linked donkey anti-rabbit IgG) and ECL-Prime Western blotting reagents were purchased from GE/Amersham. Recombinant rat PIMT was expressed and purified in our laboratory as described [27]. Recombinant human and mouse α-synucleins were purchased from ProSpec-Tany (Rehovot, Israel). Isoaspartyl delta-sleep inducing peptide (WAGGD ? ASGE, where ? designates an isopeptide bond) was purchased from Bachem.

### Mice

PIMT +/− founder mice were a generous gift of Dr. Mark Mamula (Yale University, New Haven, CT) and were originally generated by inserting a neo cassette into exon one of the *pcmt1* gene [18]. PIMT −/− (KO) and +/+ (WT) C57BL/6 mice were obtained by intercrossing PIMT +/− C57BL/6 mice. Genotyping was determined by tail DNA PCR analysis recognizing both the neo cassette and *pcmt1* gene (Transnetyx, Inc., Cordova, TN). Both PIMT KO and WT mice were sacrificed at 4–5 weeks of age.

### Preparation of mouse brain extracts

In general, mouse brains from both WT and KO PIMT genotypes were weighed immediately after removal and suspended in 9 vol of ice-cold homogenization buffer (5 mM K-Hepes, pH 7.6, 0.5 mM EDTA, 0.1 mM DTT (dithiothreitol), 10% (w/v) sucrose, and 1% (v/v) mammalian protease inhibitor mixture (Sigma)). For immunoprecipitation experiments, frozen mouse brains from both WT and KO PIMT genotypes were weighed and suspended in 5 vol of ice-cold immunoprecipitation homogenization buffer (50 mM Tris, pH 7.4, 100 mM NaCl, 5 mM EDTA, 0.3% Triton X-100, 10% glycerol, 1% (v/v) mammalian protease inhibitor mixture). In both cases, the suspension was homogenized on ice using a Potter-Elvejhem tissue homogenizer and then centrifuged at 800 ×g for 30 min. The supernatant (hereafter “brain extract") was stored at −70°C until needed.

### Two-dimensional PAGE and electroblotting

Brain extracts (200 µg protein) from both WT and KO genotypes were first diluted using homogenization buffer to a protein concentration of 2 mg/ml before being treated with TCA (to a final concentration 7% (w/v) for 1 h at 4°C. Precipitated proteins were pelleted by centrifugation at 15,800 ×g for 15 min, 4°C. Pellets were then resuspended in acetone and pelleted by centrifugation at 15,800 ×g for 5 min, 4°C. This step was repeated once before solubilizing the pellet in BioRad 2-D rehydration buffer (8 M urea, 2% (w/v) 3-[(3-cholamidopropyl) dimethylammonio]-1-propanesulfonate (CHAPS), 50 mM DTT, 0.2% (w/v) Bio-Lyte 3/10 ampholytes, 0.001% (w/v) bromophenol blue) for 1 h with gentle vortexing. The solubilized proteins were centrifuged at 15,800 ×g for 15 min, 4°C and the cleared supernatant (∼125 µl) was subjected to isoelectric focusing on IPG strips (pH 4–7 or pH 3–10 NL; 7 cm lengths) for 14,000 volt-hours at 10°C. The second dimension separation was carried out for 35 min, 200 V at 4°C on NuPage 4–12% Bis-Tris Zoom gels (Invitrogen) using MES (2-(*N*-morpholino) ethanesulfonic acid) running buffer. After electrophoresis, proteins were electroblotted onto PVDF membranes by wet transfer in 25 mM Tris, 193 mM glycine, pH 8.3, 20% (v/v) methanol at 50 V, 4°C for 3 h. After blotting, the membrane was immediately dried using 100% methanol in preparation for on-blot [^3^H]-methylation.

### On-blot methylation

On-blot methylation for detection of isoAsp-containing proteins followed the procedure of Zhu *et al.* [27]. Dried PVDF membranes holding transferred proteins were pre-wetted in 100% methanol for 30 s and rinsed in water for 2 min before blocking in 10 mM Na-MES (pH 6.2), 2 mg/ml BSA for 30 min at room temperature. After blocking, the membrane was dried in 100% methanol, placed on a glass plate and immediately covered with ∼4 ml of methylation reaction solution (100 mM Na-MES, pH 6.2, 4 µM recombinant rat PIMT, 4 µM [^3^H] AdoMet (10,000 dpm/pmol), 30% methanol, and 0.1 mg/ml BSA) warmed to 30°C. The methylation reaction was allowed to proceed for 20 min before washing the PVDF membrane twice in 10 mM Na-MES, pH 6.2, 300 mM NaCl, 0.05% Tween-20 for 10 min. Finally the PVDF membrane was washed in water for 5 min and then 100% methanol for 30 s prior to air-drying. Dried membranes were exposed to a tritium-sensitive Cyclone storage phosphor screen (Packard) for 48 h, which was then scanned on a Typhoon Trio^+^ variable mode imager (GE Healthcare). Alternatively, the dried membrane was sprayed with EN^3^HANCE (PerkinElmer) and exposed to preflashed Kodak BioMAX XAR film for 72 h at −80°C.

### Western blotting

After on-blot methylation and ^3^H-imaging, dried PVDF membranes were wetted with 100% methanol for 30 s, then water for 2 min, before blocking in 5% nonfat milk in TBS-T (Tris-buffered saline, with 0.05% (v/v) Tween-20) for 1 h at room temperature. Membranes were subsequently incubated for 90 min with primary antibodies diluted (per figure legend) in blocking solution. The membrane was then washed 3X with TBS-T followed by incubation for 1 h with goat anti-rabbit IgG conjugated to horseradish peroxidase as a secondary antibody (1∶20,000). After the membrane was washed 3X with TBS-T, the ECL-Prime Western blot detection system was used to develop the membrane. The luminescence signal was recorded using a Nikon D700 camera [29].

### Immunoprecipitation of α/β-synuclein

All steps of immunoprecipitation were performed at 4°C and all centrifugations were carried out at 720 ×g for 5 min. Brain extracts (400 µg) from both WT and KO genotypes were first diluted using homogenization buffer to a total protein concentration of 2 mg/ml before being pre-cleared with protein A-agarose (20 µl, 25% slurry) for 1 h on an end-over-end rotator. Pre-cleared supernatant was incubated with 2 µg of mouse anti- α/ß synuclein monoclonal antibody for 12 h. The antibody/antigen complex was then captured by incubation with protein A-agarose (20 µl, 25% slurry) for 1 h. The captured immune complex was pelleted by centrifugation and washed with 20 m Tris-HCl, pH 7.6, 150 mM NaCl and 1% Triton x-100 for 10 min. This wash step was repeated three times with centrifugation after each wash. After the final wash step, pelleted protein A-agarose was resuspended thoroughly in 40 µl 1X Laemmli sample buffer (125 mM Tris-HCl, pH 6.8, 10% (v/v) glycerol, 2% (w/v) SDS, 0.001% bromophenol blue) supplemented with fresh 10X NuPage reducing agent (final concentration 1X; Invitrogen) and samples were heated for 10 min at 50°C. Liberated proteins were separated from protein A-agarose by centrifugation prior to electrophoresis.

### Protein concentration

Protein determinations utilized the Pierce BCA microplate assay with bovine serum albumin as a standard.

### *In vitro* synuclein aging and isoAsp quantitation

Recombinant synucleins (0.2 mg/ml) were aged *in vitro* for 14 days at 37°C under physiological conditions (20 mM Tris-HCl, pH 7.5, 20 mM NaCl, 1 mM EDTA, 2% (v/v) glycerol, 0.05% (w/v) NaN_3_), and quantitation of isoaspartate in both unaged and aged proteins was determined by a methanol diffusion assay [30]. In brief, proteins (100 pmol) were incubated in a methylation reaction buffer (100 mM sodium phosphate, pH 6.8, 4 µM recombinant rat PIMT, 100 µM [^3^H] AdoMet (500 dpm/pmol), 5 mM EDTA and 0.2 mg/ml BSA) for 30 min at 30°C. The reaction was terminated by addition of an equal volume of stop solution (400 mM Na-borate pH 10.4, 4% SDS, 2% methanol), then 50% of the sample was transferred to an accordion-pleated filter paper lodged in a Titeseal® cap. Immediately the cap was placed onto a shell vial containing 2.5 ml of Liquiscint (National Diagnostics), and incubated for 1 h at 40°C prior to liquid scintillation counting. Isoaspartyl delta-sleep inducing peptide (50 pmol) was used as an internal standard for isoAsp quantitation.

## Results and Discussion

### Attempts to confirm isoAsp-synuclein in the PIMT-KO mouse brain

**Figure 3:**
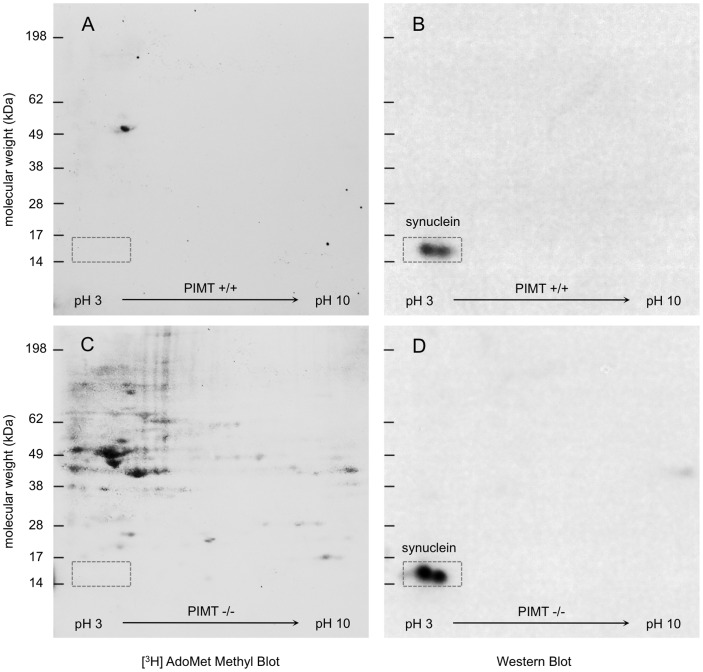
Two-dimensional protein separation and [^3^H] methyl blot analysis of PIMT wild type and knockout (mouse brain extracts using a 3–10 pH gradient in the first dimension. (A,C) Equal amounts (125 µg) of protein from each genotype were separated concurrently using 7 cm pH 3–10 non-linear IPG strips in the first dimension. After electroblotting onto PVDF, the membranes were overlaid with recombinant PIMT and [^3^H] AdoMet (4 µM, 10,000 dpm/pmol), then washed, dried and sprayed with EN^3^HANCE scintillant before performing fluorography (72 h exposure). (B,D) Western blot analysis with rabbit anti-pan synuclein (1∶8,000) of the same membranes shown in A and C.

We initiated this project by attempting to repeat a previous report [28] indicating that α- and β-synuclein are major targets for PIMT in the KO mouse brain. The results of our first attempt are shown in Fig. 3. Brain extracts of individual WT and KO mice were subjected to 2D electrophoresis utilizing pH 3–10 IPG strips in the first dimension. Isoaspartate-rich proteins were visualized by on-blot [^3^H]-methylation with purified PIMT and [^3^H] methyl-labeled S-adenosyl-L-methionine. Autoradiography revealed a large number of isoAsp-rich proteins in the KO extract, whereas only one such protein is seen in the WT. This latter protein is in the region expected for tubulin, previously identified as major methyl acceptor in our own proteomic study of PIMT targets in mouse brain [27], as well as in the Vigneswara study.

After imaging the radiolabeled isoAsp-rich proteins, the same membranes were then analyzed by Western blotting using a primary antibody that detects both α-and β-synuclein. These highly homologous proteins have molecular masses of 14.5 and 14.1 kDa, and theoretical isoelectric points of 4.7 and 4.4, respectively. A robust Western signal was seen in both the WT and KO extracts in the positions expected for the synucleins. Remarkably, we found no evidence for isoAsp-labeling in this region of either [^3^H] methyl blot.

**Figure 4:**
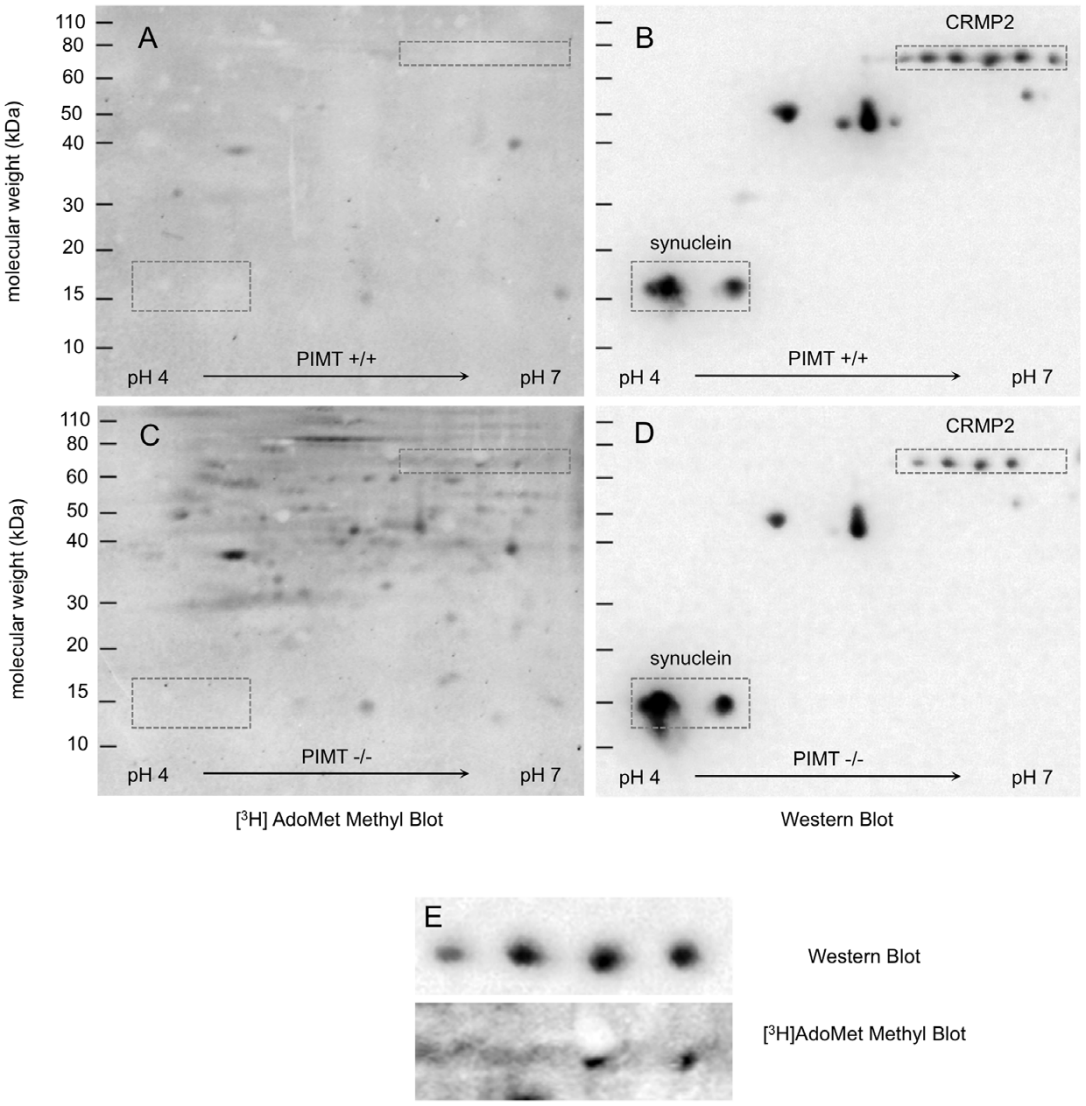
Two-dimensional protein separation and [^3^H] methyl blot analysis of PIMT wild type and knockout mouse brain extracts using a 4–7 pH gradient in the first dimension. (A,C) Equal amounts (200 µg) of protein from each genotype were separated before electroblotting onto PVDF. Membranes were [^3^H]-methylated as in Fig. 3, followed by a 48 h exposure on a tritium-sensitive phosphor-imaging screen. (B,D) Western blot analysis of the same membranes shown in A and C with a mixture of rabbit anti-pan synuclein (1∶15,000) and rabbit anti-mouse CRMP2 (1∶1,000) as primary antibodies. (E) Enhanced images of the CRMP2 region in C and D.

We next repeated this effort using a more narrow (4–7) pH gradient in the first dimension of separation (Fig. 4). Having previously shown that CRMP2 is a major target for PIMT in the KO mouse brain, we also included an antibody to this protein in the Western blot to act as an internal control. We confirmed the co-localization of CRMP2 [^3^H]-methylation and immunoreactivity, but again saw no evidence for [^3^H]-methylation of the synucleins.

**Figure 5:**
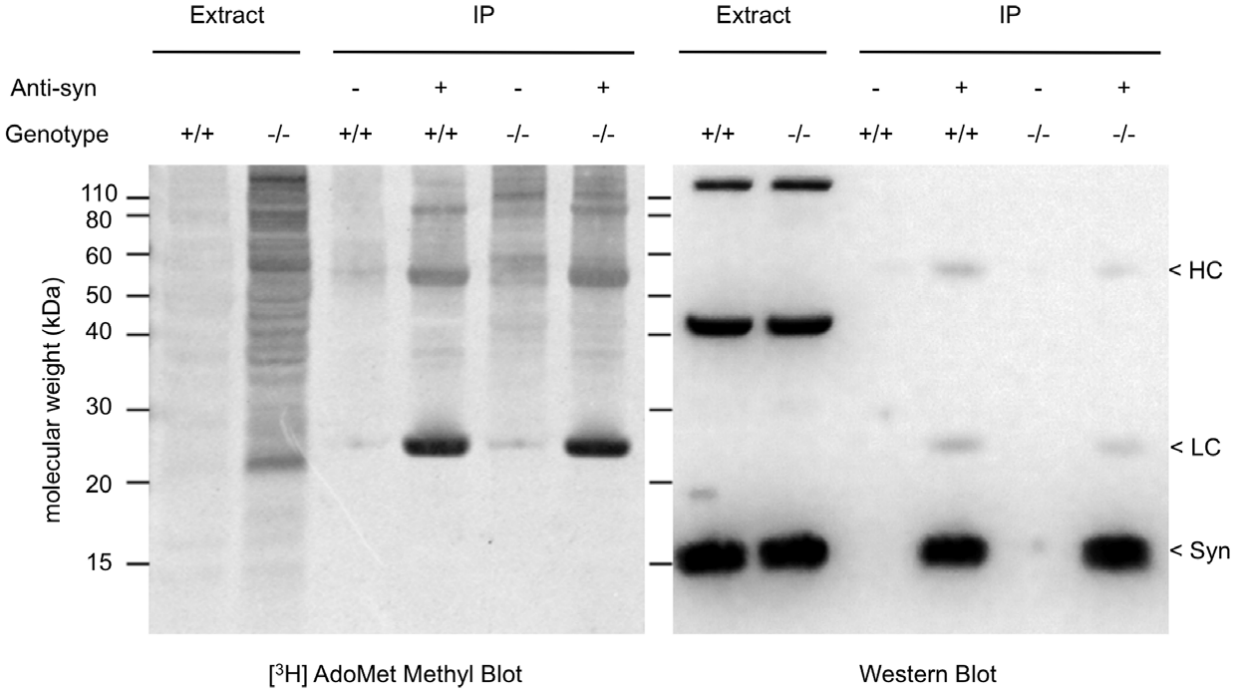
[^3^H] methyl blot and Western blot analysis of an α/β-synuclein immunoprecipitation from PIMT wild type and knockout mouse brain extracts. Equal amounts (400 µg) of protein from each genotype were immunoprecipitated with a mouse monoclonal antibody specific to α/β-synuclein. Brain extracts (20 µg per lane; lanes 1 and 2), and immunoprecipitates (lanes 3–6) were separated by 1D SDS-PAGE before electroblotting onto PVDF. The membrane was then subjected to [^3^H] methyl blot analysis as in Figs. 3 and 4, followed by a 48 h exposure to a tritium-sensitive phosphor imager screen. Using the same membrane, a Western blot (right panel) was performed with rabbit anti-pan synuclein (1∶10,000). A strong immunoreaction to α/β-synuclein was seen in lanes 4 and 6, but not in lanes 3 and 5 where the anti-synuclein antibody was omitted from the immunoprecipitation. The positions of the IgG heavy chain (HC), IgG light chain (LC), and synuclein (syn) are indicated on the far right edge. The dark bands at ∼45 kDa and ∼120 kDa in lanes 1 and 2 of the Western blot are due to proteins in the brain extract that cross reacts with anti-synuclein, similar to what is seen in panels B and D of Fig. 4.

In a final attempt to demonstrate *in vivo* isoAsp accumulation in synuclein, we carried out an immunoprecipitation in anticipation that this would increase the sensitivity of detection. α/β-Synuclein was immunoprecipitated from WT and KO mouse brain extracts and the immunoprecipitates were then subjected to SDS-PAGE. The separated proteins were transferred to PVDF, analyzed for isoAsp content by on-blot [^3^H]-methylation, and subsequently analyzed for α/β-synuclein presence by immunodetection (Fig. 5). Lanes 1 and 2 of the [^3^H] methyl blot verified increased isoAsp content in the KO extract, including a modest increase in the synuclein region at 14–15 kDa; however, immunoprecipitates from neither the WT (lane 4) nor the KO (lane 6) extracts exhibited any detectable [^3^H]-methylation in the synuclein region. The [^3^H]-methylation seen at 55 kDa and 25 kDa was expected and reflects the susceptibility of IgG heavy and light chains (present in the immunoprecipitates) to isoAsp formation [31]. A Western analysis of this blot confirmed equal amounts of synuclein in the brain extracts (lanes 1 and 2) and the immunoprecipitates (lanes 4 and 6) from the WT and KO mice. Lanes 4 and 6 also showed a light signal for the heavy and light chains of IgG owing to cross-reactivity of the goat anti-rabbit secondary antibody with the mouse monoclonal antibody to α/ß-synuclein used in the immunoprecipitation. Our interpretation of the immunopecipitation results assume that the monoclonal antibody used does not discriminate against any isoAsp form(s) of synucelin. We cannot rule this possibility out since the epitope region of the recombinant synuclein used as antigen is not reported by the supplier. However, given the size of synuclein, and the fact that the antibody was raised against the full length protein, this seems statistically unlikely. When working with polyclonal antibodies, we have never seen a reduced response to an isoAsp-bearing protein, but we have seen an enhanced response to isoAsp-proteins as predicted by the work of Mamula and coworkers [32], [33].

### Susceptibility of mouse and human α-synucleins to isoAsp formation during *in vitro* aging

As a possible explanation for the discrepancy between our synuclein results and the previous report [28], we hypothesized that synuclein might be highly susceptible to isoAsp formation *in vitro* (but not *in vivo*) and that the isoAsp accumulation seen by them might therefore be due to inadvertent protein damage in their mouse brain extracts prior to the [^3^H]-methylation step. Precedence for this is found with calmodulin, a brain-enriched protein with a mass and isoelectric point similar to that of synuclein. In the absence of calcium, calmodulin has been shown to undergo relatively rapid formation of isoAsp sites during *in vitro* aging at physiological pH and temperature [34], yet its accumulation of isoAsp in the KO mouse brain is extemely low compared to major targets such as synapsin I [35]. Indeed, calmodulin and synuclein were both among the proteins identified by Vigneswara, as major targets for PIMT in mouse brain, lending support to this hypothesis.

**Figure 6:**
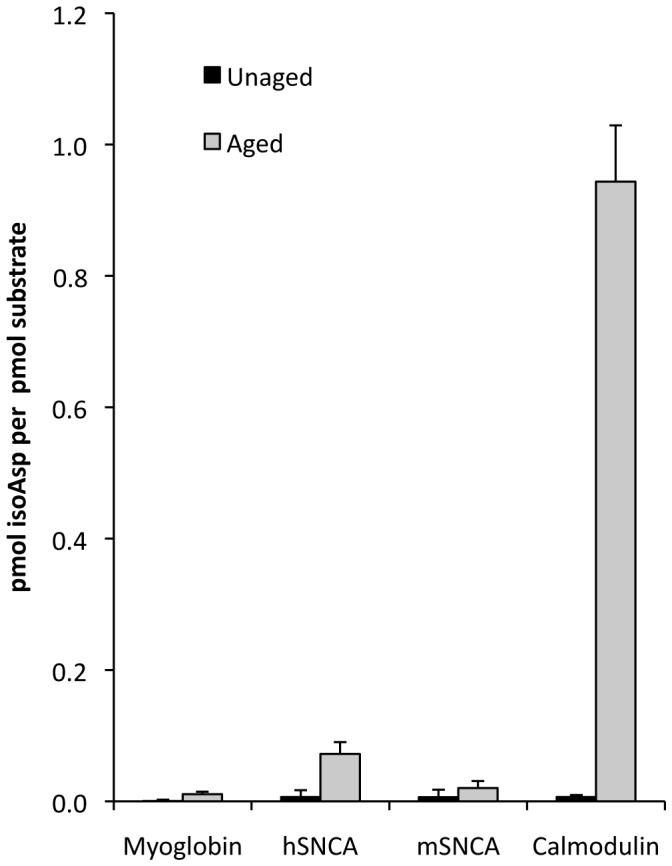
Effect of *in vitro* aging on isoaspartate accumulation in recombinant human α-synuclein (hSNCA) and recombinant mouse α-synuclein (mSNCA). Aging was carried out at pH 7.5, 37°C for 14 days. Isoaspartate accumulation was measured by a methanol diffusion assay. Horse heart myoglobin and bovine brain calmodulin were included as controls for proteins that are known to be highly resistant, or highly susceptible, respectively, to isoaspartate formation when aged under these conditions. Each assay was performed in triplicate. *Error bars* represent standard deviation.

To test this idea, we made use of the fact that purified recombinant α-synucleins of both mouse and human origins are commercially available. These synucleins were subjected to *in vitro* aging for two weeks at 37°C and then assayed for isoAsp content. Parallel experiments were carried out with myoglobin (which is highly resistant to isoAsp formation) as a negative control, and calmodulin as a positive control. As shown in Fig. 6, both human and mouse α-synucleins accumulated isoAsp during *in vitro* aging to a level that was slightly greater than myoglobin and much less than calmodulin. This is consistent with the fact that neither α- nor ß-synuclein contain an Asx residue in a hot-spot sequence wherein the C-flanking amino acid is a Gly, Ser, or His. The very low rate of isoAsp accumulation seen in mouse α-synuclein (1/40th that of calmodulin) argues against the idea that extensive pre-methylation protein damage was responsible for the identification of synuclein as a major PIMT target by Vigneswara. The somewhat higher level of isoAsp accumulation in human *vs*. mouse α-synuclein is likely due to sequence differences at positions 103 and 122 (Table 1). These are the two most deamidation-prone Asn residues in human α-synuclein [36] and are replaced by non-Asx residues in the mouse sequence.

**Table 1:** Parameters of all Asn and Asp sites in mouse and human α-synuclein.

AA#	Sequence	Mus	Hum	Deamidation half-life @ pH 7.4, 37°C^a^ (*days*)	Local Flexibility^b^
02	MDV	X	X	-	
65	TNV	X	X	237	.685/.685
87	GNI	X	-	287	.583/-
98	KDQ	X	X	-	
**103**	**KNE**	-	X	**72.5**	**-/.762**
115	EDM	X	X	-	
119	VDP	X	X	-	
121	PDN	-	X	-	
**122**	**DNE**	-	X	**46.8**	**-/.696**
135	QDY	X	X	-	
All					.625/.619
	GNG			1.0^c^	
	GNS			11.8	
	GNH			9.2	

a Half-lives are those reported for synthetic pentapeptides containing the trimer sequence shown, and bounded by a glycine at each end [6]. Values for the two Asn residues unique to the human sequence are bold-faced.

b Average flexibilities for mouse and human sequences were calculated using ProtScale at www.ExPASy.com with a 7-residue moving window and normalization to a scale of 0–1.

c Same as (a) above for three widely reported hot-spot sequences in peptides and proteins.

### Why two different proteomic studies generated widely different profiles of isoaspartyl protein accumulation in the same PIMT knockout mouse model

In their 2D gel-based proteomic analysis of isoaspartyl proteins in the PIMT knockout mouse, Vigneswara et al. [28] used peptide mass fingerprinting to identify 11 distinct polypeptides ranging in molecular weight from 14 kDa (α- and ß-synuclein) to 199 kDa (MAP-2). Our parallel analysis in the same mouse model identified 22 distinct polypeptides ranging in molecular weight from 29 kDa (carbonic anhydrase II) to 97.8 kDa (dynamin-1) [27]. There were only two polypeptides that were found in both studies; α- and β-tubulin. Since the same mouse model was used in both studies, the divergent results must stem from different methodological approaches used by these two groups to separate and identify the proteins. In the Vigneswara study, brain extracts were ^3^H-methylated by PIMT and then subjected to a series of steps that included precipitation with trichloroacetic acid, dissolution in an IPG rehydration buffer (pH 6.1), adsorption into an IPG (pH 4–7) strip in that same buffer, isoelectric focusing, equilibration of the IPG strip with a second dimension SDS sample buffer (pH 6.8), second dimension SDS-PAGE, and finally transfer at pH 7.2 onto a PVDF membrane, prior to imaging the tritium signals on a highly sensitive microchannel plate detector. Given that protein isoaspartyl methyl esters in hotspot sequences can have a half-life as short as 7 min at pH 7.4, 37°C [10], it was of interest to estimate how the above procedures would influence the final profile of ^3^H-methylated proteins at the stage of image acquisition.

**Figure 7:**
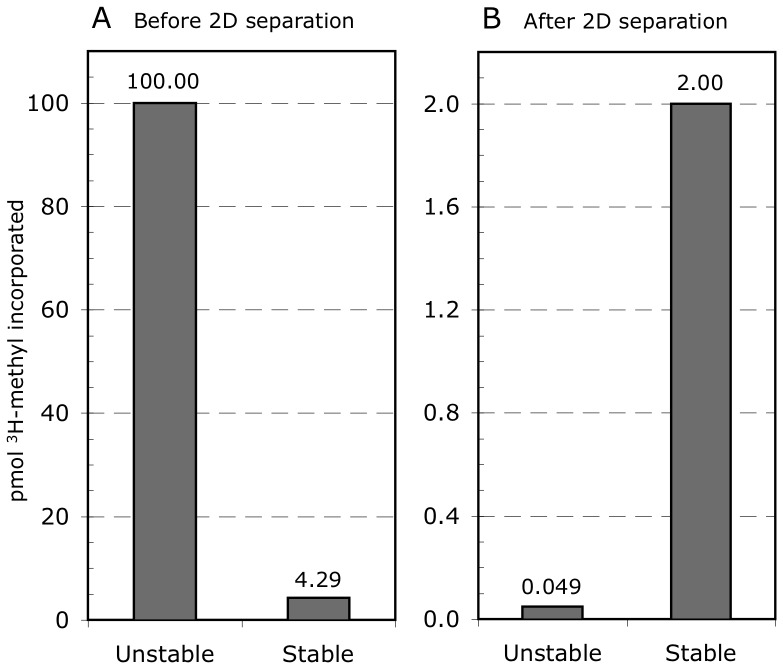
Modeling of the survivability of unstable *vs* stable substrates for PIMT. Panel A shows the expected relative abundance of isoAsp sites (judged by methyl-accepting capacity) as it would occur in PIMT knockout mouse extracts immediately after ^3^H-methylation of isoAsp sites by PIMT. Panel B shows the relative abundance of these two classes of isoAsp sites after being subjected to analytical procedures for 56 h under conditions where the two classes have average half-lives of 300 and 3000 min, respectively. Note the different scales used in panels A and B.

Fig. 7 shows the results of a simulation designed to model the relative recoveries of ^3^H-methyl esters of the unstable and stable classes of PIMT substrates after the 2D separation and blotting procedures used by Vigneswara. The simulation assumes that (a) the relative amount of unstable (succinimide-prone) and stable (succinimide-resistant) protein methyl esters is 100 pmol and 4.2 pmol immediately after the ^3^H-methylation step (Fig. 7A), (b) the average half-life of these methyl esters is 300 and 3000 min respectively, and (c) the procedures lasted 56 h, during which the methyl esters were susceptible to hydrolysis at varying rates. We assumed an average half-life for the unstable class of 300 min, rather than 7.2 min because the Vigneswara procedures were designed to minimize hydrolysis by keeping pH and temperature as low as practical within the limits of the procedures used, and some of the detergents used are known to stabilize isoaspartyl methyl esters. As a point of reference, we have observed that the isoAsp methyl esters of aged calmodulin have a half-life of about 2 hr (120 min) under conditions of SDS-PAGE using the same gel and buffer system employed by Vigneswara et al. (C. David and D. Aswad, unpublished). The 56 hr duration was gleaned from the details of the Vigneswara methods section, along with our own experience with the procedures used. The half-lives for each class were converted to first-order rate constants and used to calculate the surviving ^3^H-methyl label using the equation A/A_o_ = e^−kt^ (Fig. 7B). It is clear that the profile of ^3^H-methyl esters shifts dramatically during the 56 h simulated analysis procedure such that the less abundant stable class dominates the profile by a factor of 41 to 1. This is because the predicted loss of the unstable (succinimide-prone) class on methyl-esters is 99.95%, wheras the loss of the stable class is only 53.4%. If we double the half-life of the unstable class to 600 min, the loss is still large at 97.8%. There is no question that long-duration analytical procedures employed with the unstable class of isoaspartyl methyl esters entail massive losses that dramatically biases the final profile of endogenous PIMT substrates.

We maintain that the simulation results above provide a cogent explanation as to why two different proteomic studies generated widely different profiles of isoaspartyl protein accumulation in the same PIMT knockout mouse model. Our methodology used an on-blot ^3^H-methylation procedure in which proteins were first separated by 2D electrophoresis and blotted onto PVDF before the ^3^H-methylation step, such that the exposure of the methyl esters to hydrolysis conditions was limited to a few minutes at pH 6.2 and room temperature. Because of the overall predicted loss of ^3^H label during the Vigneswara procedure, it is impressive that they were able to obtain the signals that they did. This can be attributed to their use of a microchannel plate detector; a highly sensitive imaging device for autoradiography that is not widely available [37].

The lability of isoaspartyl protein methyl esters, though not always appreciated, has facilitated research on the on the formation, repair, and localization of isoaspartyl sites in polypeptides. Lindquist and McFadden ([38]) carried out PIMT-dependent methylation and spontaneous demethylation in the presence of O^18^ water to show that only one O^18^ is incorporated for each cycle of succinimide hydrolysis, proving that efficient repair of isoAsp linkages requires multiple cycles through the repair pathway shown in Fig. 1. In the same vein, Liu et al. ([39]) showed that this selectivity of O^18^ incorporation, in conjunction with mass spectrometry, can unambiguously assign the site(s) of isoAsp formation in a polypeptide with multiple Asx residues. PIMT-dependent priming of isoAsp sites to promote succinimide formation has also been used to selectively cleave isoAsp linkages in peptides sites using hydroxylamine ([40]), or to incorporate hydrazine as the first step in affinity column enrichment of isoAsp proteins or in the identification of isoAsp sites *via* mass spectrometry ([41]).

## Conclusions

(1) α- and β-synuclein are relatively resistant to isoAsp formation both *in vivo* and *in vitro* and should not be considered as major targets for the PIMT repair enzyme. Therefore, it is unlikely that isoAsp formation plays any significant role in the etiology of synucleinopathies.
(2) When attempting to profile the major targets for PIMT, it is important to use procedures that avoid an unintended enrichment for minor substrates that typically harbor less abundant, yet more stable, isoAsp sites.
